# A nationwide cohort study on pneumonia infections among agriculture and healthcare workers in Taiwan

**DOI:** 10.1017/S0950268824001304

**Published:** 2024-12-05

**Authors:** Erik Pieter de Jong, Chi-Hsin Sally Chen, Wei-Chi Lin, Chia-Yu Chang, Chang-Chuan Chan

**Affiliations:** 1 Global Health Program, College of Public Health, National Taiwan University, Taipei, Taiwan; 2 Institute of Environmental and Occupational Health Sciences, College of Public Health, National Taiwan University, Taipei, Taiwan

**Keywords:** bacterial infections, pneumonia infection, risk assessment, agricultural workers, healthcare workers, occupation-related infections, claim data, public health, occupational health

## Abstract

Bacterial infection risk in work environments has been extensively reported for healthcare workers, while this risk is rarely researched in other occupations. This study aimed to identify occupational environments in Taiwan’s agricultural and healthcare industries with elevated bacterial infection risks by comparing risks for general bacterial infections and pneumonia. Using labour and health insurance claim data from 3.3 million workers (January 2004–December 2020), a retrospective cohort was constructed to estimate occupational infection risks with Cox regression and the Anderson-Gill extension. Significantly elevated hazard ratios were found for workers in vegetable growing, crop cultivation service, mushroom growing, flower growing, and fruit growing, ranging from 1.13 to 1.39 for general bacterial infections and 1.68 to 3.06 for pneumonia infections. In afforestation and the inland fishing industry, pneumonia risk was significantly elevated with, respectively, 1.87 and 1.21. In the healthcare section, especially workers in residential care services and residential care services for elderly stand out regarding their pneumonia risk, with significant hazard ratios of 3.49 and 1.75. The methods used in this study were proven to be effective in identification of occupation environments at risk and can be used in other settings. These findings call for prioritization of bacterial infection prevention by occupation.

## Key findings

- Workers in occupational environments in close contact with soil, as well as inland fishing have significantly higher risks for pneumonia infections.
- Besides hospital settings, workers in residential care services in Taiwan showed a significantly elevated pneumonia and general infection risks.
- This study’s innovative methods proved to be a successful surveillance tool to identify occupational environments with increased risks of bacterial infections.

## Introduction

Bacterial infections are threatening health globally, especially since they become harder to treat due to antimicrobial resistance (AMR). Already in 2015, the World Health Assembly adopted the Global Action Plan on AMR, outlining core actions against bacterial drug-resistant infections [1]. Reducing bacterial infections all together regardless of their antibiotic resistance status is at the core of this plan, since prevention of bacterial infections also prevents resistant infections [1, 2].

Effective prevention of bacterial infections should be seen through a One Health lens, as humans, animals, and the environment all play a vital role in the transmission chain. This environmental component within One Health is often neglected in research, while it is contributing to meaningful differences in vulnerability [3, 4]. Time spend in contaminated environments, for example, is seen as an individual determinant for bacterial infection risk, with work environments as an overarching determinant for bacterial infection risk [5, 6]. The recent COVID-19 pandemic, for example, has highlighted the importance of proper infectious disease protection for workers beyond healthcare personnel, as workplaces proved to be environments in which transmission flourished [7–9]. This also applies to bacterial infections as most workers spend a significant portion of their day in shared work environments, where they closely interact with co-workers, handle pathogen-rich materials, or share their workspace with animals [5, 10]. Haagsma *et al*. [11], for example, concluded in a systematic review that people working in the healthcare industry and agricultural industry environments had an increased exposure risk and high infection risk. Chung *et al.* [12] came to a similar conclusion by analysing Korean occupational injuries and disease reports, but also identified workers in the fishing industry to be at risk for infection. Their research linked 22 deaths over a 9-year period to occupational infection, a large share of these infections was caused by bacteria [12]. Research specifically focusing on healthcare workers in the United States, estimated 9–42 deaths per million healthcare workers was related to bacterial and viral infections such as *hepatitis B*, *hepatitis C*, *tuberculosis, or HIV* [13]. Although these figures show the serious health threat that occupational bacterial infections entail for specific occupation sections, a comprehensive comparison of such risk for workers is lacking. This is troubling prioritization of counter measures for occupations with elevated bacterial infection risks.

This study was the first of its kind to construct a nationwide retrospective bacterial infection cohort based on bureau of labour insurance (BLI) and national health insurance (NHI) claim data in Taiwan. By utilizing complex data available in both datasets, occupational bacterial infection risk could be assessed and compared. The studies’ primary objective was to assess general bacterial infection risk among workers’ industrial classes within agricultural and healthcare environments, and compare their risk for bacterial infections with a large reference group. The secondary objective of this study was to comparatively assess each occupational class’s risk for pneumonia, a bacterial infection that has been ranked as the third leading cause of death in Taiwan [14]. For both objectives, Cox proportional hazards modelling was used in combination with the Andersen-Gill (AG) extension, to test the hypothesis that individual occupational classes are not different in their risk for, respectively, general bacterial infection or pneumonia infections, in comparison to the reference group [15, 16]. With the results of this research, prevention can prioritize occupational classes most at risk.

## Methods

### Study design and settings

The Institute of Labor, Occupational Safety and Health, under the Ministry of Labor of Taiwan, provided labour insurance data for each month from January 2004 to September 2021 (16 years and 9 months) at the individual’s level. From these data, the first 192 months of data were used to match the same timeframe of the NHI data. Each employing entity, such as companies and institutions, is registered in the Labor Insurance Institutional database and categorized into industry sections and classes, following a system similar to the International Standard Industrial Classification of All Economic Activities (ISIC). This classification system is structured with consecutively sections, divisions, groups, and classes, where 21 sections are large overreaching categories, while classes entail the most detailed level of economic activity [17]. Individuals were included in the initial cohort selection when they were affiliated one of the following six industrial sections: (1) Section A – Agriculture, Forestry, Fishing, Animal Husbandry; (2) Section Q – Human Health and Social Work; (3) Section F – Construction; (4) Section K – Finance and Insurance; (5) Section L – Real Estate; and (6) Professional, Section M – Scientific, and Technical Service. The agriculture, forestry, fishing, animal husbandry section, and the human health and social work section were considered as the exposure group in this study. Literature has occasionally indicated a higher exposure among workers in these sections, as well as a higher infection risk due to the nature of the work conducted in these industries [10–12, 18]. How workers in classes within these two industrial sections compared with each other was, however, unknown before this study. The other industrial sections were selected according to their perceived lower risk for exposure and infections and formed the reference group [10–12, 18].

The initial selection of individuals from the labour insurance data set resulted in a cohort of 9.1 million (9,101,740) individuals, for both the exposure group and reference group combined. After the initial selection, individuals with missing or conflicting information about their birthdays were excluded. In addition, exclusions were based on the quality of the BLI data, where some individuals had invalid insurance wages that were excessively high or negative. Furthermore, people were excluded if their age was invalid, if their recorded national ID did not match the NHI database, or if their death did not match with their BLI insurance timeframe. Some selected individuals also worked multiple jobs (resulting in insurance periods with different companies); this could be within the same industrial class or in another industrial class. Due to the study’s design where industrial class is an exclusive classification parameter, individuals who worked multiple jobs in different industry classes were therefore excluded. Individuals who worked multiple jobs within the same industrial class were included in the cohort, as a combined record per individual.

### Data sources and variables

**Figure 1.**
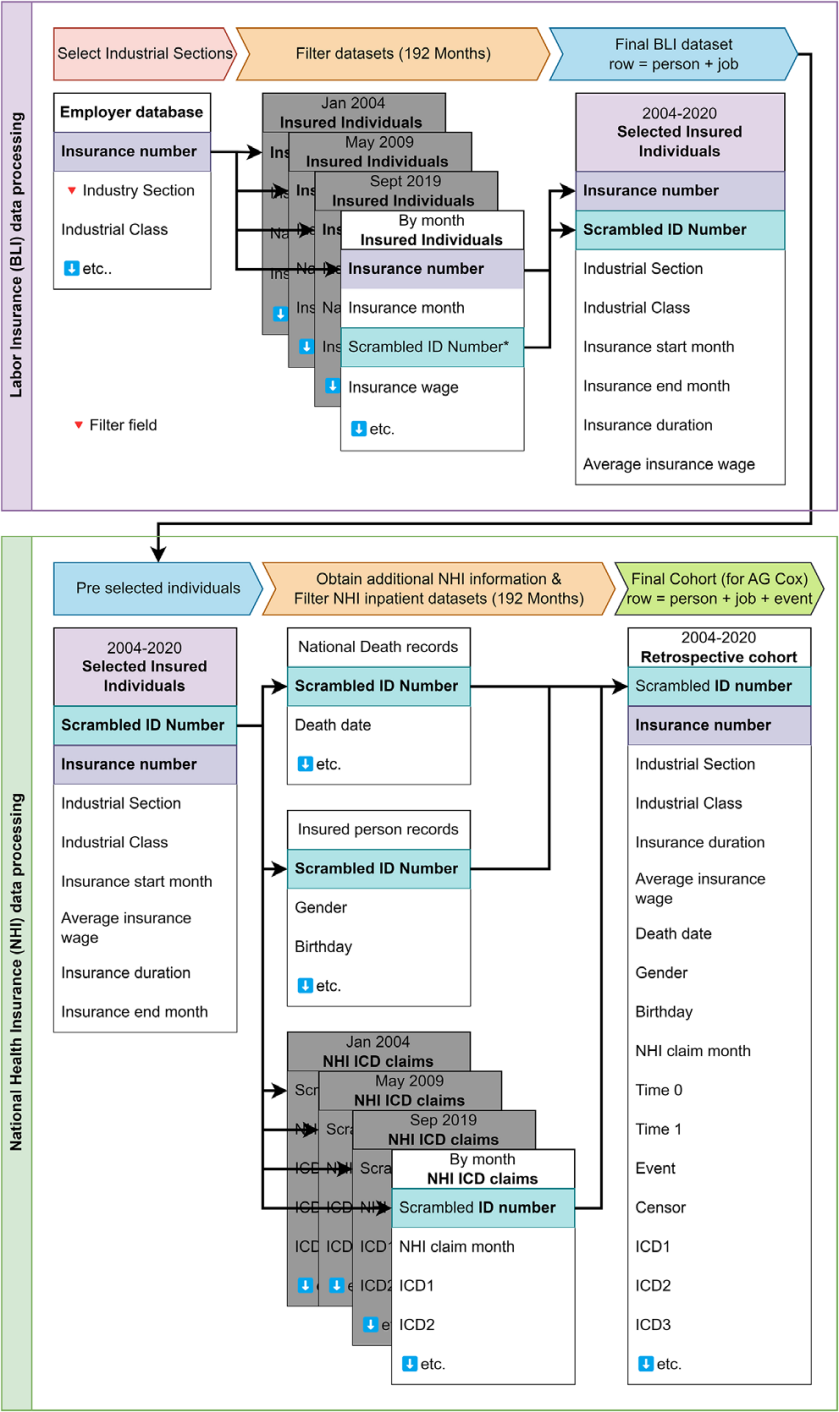
BLI and NHI cohort data processing steps (flowchart). Flowchart describing the process to combine Taiwan’s national labour insurance and national health insurance databases and records for January 2004 until December 2020 (included).

Figure 1 shows the data processing steps carried out in this study to construct the cohort suitable for AG Cox regression. In Figure 1, insurance number and scrambled anonymized national ID number are the unique identifiers, used to connect individual employment insurance information with employer information and individual health claim records. The anonymization of personal data was carried out by the labour insurance organization in close collaboration with the NHI. All data handling was carried out with SAS 9.4 in the Ministry of Health and Welfare’s secured datacentre, to protecting privacy sensitive data. The research proposal and protocol were reviewed by the institutional review board and approved under number 202112HM017.

#### BLI data

A list of companies and institutions in the industrial sections of interest was generated from the BLI data using the industry section as a filter (Figure 1, employer database). This list was used to filter and link all individual BLI records by month from January 2004 to December 2020 (192 months) based on insurance numbers (highlighted in Figure 1). The final dataset contained one row per insured individual, including their deidentified ID number, company/institution number, insurance start and end months, insurance duration, and mean insured wage (Figure 1, selected insured individuals).

#### NHI data

The claim data provided by NHI spanned from January 2004 up to and including December 2020, and contained 192 monthly files with nationwide inpatient claim records (Figure 1, NHI ICD claims). The international classification of disease (ICD), either ICD-9CM or ICD-10CM, depending on the timeframe, is used within these claim records. A comprehensive list of ICD codes was developed by combining Patkar et al.’s [19] ICD-9-CM sensitive list for bacterial infections, Schneeweiss et al.’s [20] ICD-9-CM list of serious bacterial infections, and Hashimoto et al.’s [21] ICD-10-CM adaptation of Fleming-Dutra et al.’s [22] list of bacterial infection diagnoses where antibiotics are indicated for treatment. These ICD codes were mapped according to their ICD versions and checked for their relevance in Taiwanese clinical settings, the resulting ICD list can be found in the Supplementary Materials. After filtering by ICD, a dataset was formed containing all bacterial infection events for the cohort, together with the event date. Simultaneously, birthday, gender, and if applicable death date, were obtained for all individuals in the cohort from NHI insured person records and national death records. The final step of the process schematically depicted in Figure 1 was the formation and restructuring of the obtained dataset, to facilitate AG Cox regression. A similar process was used to generate a cohort based on just pneumonia due to unspecified organism (ICD-9-CM 486, ICD-10-CM J18.9 and corresponding ICD-9-CM 486 Both ICD codes for pneumonia, version difference between ICD-9-CM and ICD-10-CM). With these two generated cohorts, AG Cox regression analysis was carried out for the two defined outcomes, general bacterial infection risk and pneumonia infection risk.

### Statistical methods

The AG extension to the proportional intensity model makes it possible to apply a survival model to recurring events. It assumes that subjects have the same risk for developing events over time and therefore that recurring events within the individual do not influence one another. To overcome the violation of the independent observations’ assumption, sandwich variance estimate parameters were calculated. By calculating sandwich variance estimates, possible heteroscedasticity is better accounted for in the calculation of regression coefficients [23, 24]. SAS 9.4 PHREG procedure was used to calculate adjusted hazard ratio (aHR) together with 95% confidence intervals (CIs), for each industrial class in the agricultural or healthcare section that contained over 1,000 individuals and had a clearly defined ISIC class. The models were adjusted by pre-defined variables such as gender, age at time of event, average insurance wage and average insurance duration.

## Results

### Cohort demographics

A combined total of 3.3 million individuals were insured in the industry sections of interest eligible for inclusion in the retrospective cohort. As shown in Table 1 the healthcare section (agriculture, forestry, fishing, animal husbandry) included 284,837 individuals, of which the majority was female. Their age at the start of the study was 51 years and the largest share was over 60 years old. At age 54.5, this group had their first bacterial infection and their most recent bacterial infection at 58.9 years of age. The follow-up time for the agricultural group was 87.9 months on average, insurance duration for this group was 69.7 months. On average the wage on which the individual’s labour insurance was calculated was 23,099 New Taiwan Dollar (NTD) per month, similar to 740 USD (in January 2024). Individuals in the agricultural section on average went through 9.4 bacterial infection during the study.

**Table 1.** Basic cohort demographics at baseline and during events

Variable categories	Overall (*n* = 3,256,491)		Section A (*n* = 284,837)		Section Q (*n* = 404,707)		Reference sections (*n* = 2,732,493)	
Gender								
Male	1,347,123	(41.4%)	102,583	(36.0%)	77,895	(19.2%)	1,166,645	(45.4%)
Female	1,385,370	(42.5%)	138,645	(48.7%)	242,890	(60.0%)	1,003,835	(39.1%)
Unknown	523,998	(16.1%)	43,609	(15.3%)	83,922	(20.7%)	396,467	(15.4%)
Age at baseline (years)								
Average (years, SD)	39.0	± 15.3	51.0	± 14.7	36.0	± 13.9	38.2	± 15.0
<30	1,563,629	(48%)	73,809	(25.9%)	225,489	(55.7%)	1,264,331	(49.3%)
30–39	514,541	(16%)	28,944	(10.2%)	70,216	(17.3%)	415,381	(16.2%)
40–49	345,036	(11%)	28,696	(10.1%)	42,544	(10.5%)	273,796	(10.7%)
50–59	441,789	(14%)	60,218	(21.1%)	39,967	(9.9%)	341,604	(13.3%)
>60	391,496	(12%)	93,170	(32.7%)	26,491	(6.5%)	271,835	(10.6%)
Age at first bacterial infection (years, SD)	42.5	± 16.0	54.5	± 15.2	39.1	± 14.7	41.7	± 15.7
Age at last bacterial infection (years, SD)	47.2	± 16.0	58.9	± 15.0	43.5	± 14.9	46.5	± 15.7
Follow-up time (months, SD)	92.9	± 60.4	87.9	± 59.0	85.0	± 60.4	94.7	± 60.5
Insurance duration (months, SD)	34.4	± 45.2	69.7	± 56.8	30.3	± 39.4	31.2	± 42.9
Insurance wage (TWD, SD)	24,213	± 9,129	23,099	± 6,740	23,814	± 9,067	24,400	± 9,355
Average number of bacterial infections (N, SD)	8.4	± 15.8	9.6	± 18.7	7.5	± 14.0	8.4	± 15.6

Section A, Agriculture, Forestry, Fishing and Animal Husbandry; Section Q, Human Health and Social Work; Reference sections, Construction, Finance and Insurance, Real estate and Professional, Scientific and Technical Service combined; SD, Standard Deviation; TWD, New Taiwan Dollar currency.

For the healthcare section (human health and social work, n = 404,707), the proportion females was larger and the average age at the start was 15 years younger, with 36 years. The majority of this group was aged under 30 years old, and on average, their first event took place at age 39.1 and their last event in this cohort at age 43.5. Follow-up time was similar to the agricultural section with 85 months, but their insurance duration was much shorter with 30.3 months. This group had a slightly higher average wage with 23,814 NTD. On average, during follow-up in this study, workers in the healthcare industry went through 7.5 bacterial infection events.

The combined reference group (n = 2,732,4,493) was more equally distributed regarding gender. Ages on average were similar to the healthcare section. Follow-up time for the reference group was slightly longer, with 94.7 months on average. The average number of events was in-between those of the agricultural and healthcare section.

### General bacterial infections and pneumonia

Table 2 provides an overview of the prevalence of general bacterial infections and pneumonia per occupational environment over the follow up of this study. For industrial classes in the agricultural and healthcare section, general bacterial infection prevalence ranged from 290 to 3,667 per 100,000 individuals, while for pneumonia the prevalence ranged from 142 per 100,000 to 1,617 per 100,000. When looking at specific industrial classes, especially afforestation (1617), fruits growing and fruit tree cultivation (1028), marine fisheries (969), pig farming and pig breeding (819), and Inland Fishing Industry (808) have a relatively high number of pneumonia prevalence per 100,000. Within the Human Health and Social Work section, both industry classes related to residential care are noticeably higher in their pneumonia prevalence (500 and 706 per 100,000).

**Table 2.** General bacterial infection and Pneumonia prevalence by industry section and class, 2004–2020 (infections per 100.000)

Industry section and industry class	All bacterial infections^a^	Pneumonia^b^
*Agriculture, Forestry, Fishing, Animal Husbandry*		
Vegetable growing and vegetable cultivation	290	290
Crop cultivation service	1,394	796
Mushrooms growing and edible mushroom cultivation	599	261
Flower growing and floriculture	687	289
Fruits growing and fruit tree cultivation	1,480	1,028
Post-harvest processing of crops	662	506
Afforestation	3,667	1,617
Inland fishing industry	2051	808
Livestock service industry	463	252
Mariculture	1,259	748
Marine fisheries	2,208	969
Chicken farms and chicken breeding	1,028	514
Pig farms and pig breeding	1,387	819
*Human Health and Social Work*		
Clinics	740	201
Hospital	818	186
Residential care services	500	500
Residential care for the elderly	1,335	706
Social work services for people with physical and mental disabilities	1,027	375
Social work services for children and youth	544	142
Medical test laboratory and medical laboratory services	413	286
*Reference group*	1,245	477

a ICD codes defined in Supplementary Materials.

b Organism unspecified (ICD-9-CM: 486; ICD-10-CM: J18.9) + ICD-9-CM: 486 Both ICD codes for pneumonia, version difference between ICD-9-CM and ICD-10-CM.

### Risk assessment

The outcomes of the multivariable model for general bacterial infection and pneumonia risk can be found in Table 3. For the agricultural section, aHRs for general bacterial infections range from 1.39 (95% CI 1.24–1.55) for people employed in vegetable growing to 0.94 (95% CI 0.91–0.97) for workers in the inland fishing industry. Industrial classes related to soil such as vegetable growing, crop cultivation service, mushroom growing, flower growing, and fruit growing all have significantly elevated aHRs for general bacterial infections. aHRs were also elevated significantly for people working in the livestock service industry class and mariculture class regarding general bacterial infections. A significantly lower hazard ratio (HR) was reported for both the inland fishing industry class and the marine fishing class. The risk for pneumonia infections, a more specific outcome, was also the highest for people working in the vegetable growing class, with an aHR for pneumonia of 3.06 (95% CI 1.0–8.55). The lowest significant aHR for pneumonia infections for this section was for people working in post-harvest processing of crops, followed by the marine fishing industry class. The earlier pointed out industrial classes related to soil, all had significantly elevated aHRs for pneumonia infections, for example, workers in crop cultivation showed a 2.02 (95% CI 1.40–2.93) times higher risk. Afforestation, an occupational class linked to soil as well, had an elevated aHR of 1.87 (95% CI 1.47–2.38) for pneumonia. For employees in the inland fishing industry, their risk for pneumonia infections was significantly elevated with an aHR of 1.21 (95% CI 1.13–1.29), in contrast to their lower risk for general bacterial infections.

**Table 3.** Multivariable risk analysis by industry section and class, using AG Cox regression, for general bacterial infections and pneumonia

	Multivariable AG Cox models	
	General bacterial infections^a^	Pneumonia^b^
Industry section and industry class	aHR (95% CI)	aHR (95% CI)
*Agriculture, Forestry, Fishing, Animal Husbandry*		
Vegetable growing and vegetable cultivation	1.39 (1.24–1.55)**	3.06 (1.10–8.55)*
Crop cultivation service	1.22 (1.15–1.30)**	2.02 (1.40–2.93)**
Mushrooms growing and edible mushroom cultivation	1.28 (1.22–1.35)**	1.40 (1.01–1.92)*
Flower growing and floriculture	1.19 (1.15–1.24)**	1.68 (1.28–2.21)**
Fruits growing and fruit tree cultivation	1.13 (1.03–1.23)*	1.68 (1.22–2.31)*
Post-harvest processing of crops	1.01 (0.94–1.09)	0.46 (0.28–0.73)*
Afforestation	1.05 (0.99–1.12)	1.87 (1.47–2.38)**
Inland fishing industry	0.94 (0.91–0.97)**	1.21 (1.13–1.29)**
Livestock service industry	1.23 (1.13–1.34)**	1.63 (0.93–2.85)
Mariculture	1.10 (1.01–1.18)*	1.31 (0.87–1.97)
Marine fisheries	0.95 (0.92–0.98)**	0.91 (0.84–0.99)*
Chicken farms and chicken breeding	1.03 (0.97–1.09)	1.13 (0.87–1.46)
Pig farms and pig breeding	0.97 (0.93–1.01)	1.20 (0.94–1.54)
*Human Health and Social Work*		
Clinics	1.12 (1.11–1.14)**	0.98 (0.89–1.07)
Hospital	0.94 (0.92–0.95)**	1.20 (1.12–1.29)**
Residential care services	2.27 (1.85–2.77)**	3.49 (1.51–8.08)*
Residential care for the elderly	1.13 (1.09–1.18)**	1.75 (1.48–2.06)**
Social work services for people with physical and mental disabilities	1.09 (1.05–1.13)**	1.24 (1.02–1.50)*
Social work services for children and youth	0.98 (0.96–0.99)*	0.81 (0.71–0.92)*
Medical test laboratory and medical laboratory services	1.06 (0.99–1.14)	0.94 (0.65–1.37)

a ICD codes defined in Supplementary Materials.

b Organism unspecified (ICD-9-CM: 486; ICD-10-CM: J18.9).

* *P*-value <0.05.

** *P*-value <0.0001.

aHR, adjusted hazard ratio; CI, confidence interval.

In the healthcare section, general bacterial infection aHRs range from 2.27 (95% CI 1.85–2.77) for workers in residential care services, to 0.94 (95% CI0.92–0.95) for workers in hospital settings. Personnel in the clinic class showed a significantly elevated aHR of 1.12 (95% CI 1.11–1.14) for general bacterial infections and workers in residential care for the elderly had a general bacterial infection risk that was 1.13 (95% CI 1.09–1.18) times higher compared to the reference group. People who were employed in the social work services for people with physical and mental disabilities class had a significantly elevated aHR for bacterial infections as well, while the social work services for children and youth class had a lowered aHR. For the more specific outcome, pneumonia infections, the highest aHR was reported for the residential care services class with 3.49 (95% CI 1.51–8.08) and for the residential care for the elderly class with 1.75 (95% CI 1.48–2.06). Workers in hospital settings were 1.20 (95% CI 1.12–1.29) times as likely to develop a pneumonia infection, compared to the reference group. An aHR of 1.24 (95% CI 1.02–1.50) for workers in social work services for people with physical and mental disabilities also stands out, while social work services for children and youth had a lowered aHR of 0.81 (95% CI 0.71–0.92).

## Discussion

This research identified pneumonia due to an unspecified pathogen as the most predominant bacterial infection among hospitalized individuals who were insured in the agriculture section or healthcare section between 2004 and 2021. Previous research shows that pneumonia is one of the main reasons for hospitalization and is more common than often believed [25]. In Taiwan, pneumonia was ranked as the third cause of death in 2017, with this study adding insights to the threat pneumonia entails for workers in specific industries and their thereby their families [26]. In the results of the regression models for the specific pneumonia outcome, working environments related to soil stand out with vegetable growing, crop cultivation service, mushroom growing, flower growing, fruit growing, and afforestation classes, all showing significantly elevated HRs for pneumonia due to unspecified organisms. Research in other countries has shown that especially vegetable production and crop cultivation is prone to bacterial contamination of produce and soil, when contaminated water is used in irrigation or when livestock manure is used in fertilization [27, 28]. In addition, it has been argued that workers throughout food production can become carrier of bacteria and thereby play a key role in household, consumer and community transmission of bacterial pathogens [28]. The significantly elevated risk for other soil related occupational environments besides crop cultivation and vegetable growing for specifically pneumonia, as reported in this study, underline the importance of protection of workers across all these environments. With relatively simple and cost-effective measures such as improved handwashing and the use of gloves for example, workers in soil related environments can be better protected and it potentially protects individuals further down the transmission chain such as family members [18]. Employees in post-harvest processing of crops in the Taiwanese setting showed a noticeable lower and significant aHR for pneumonia in this research. This is an industry that is known to have implemented such improved hygiene practices to prevent cross contamination of produce that would harm consumers.

From the same specific model, the inland fishing industry stands out, which is an industry that is important in the Taiwanese context. Workers in this industry showed a significantly higher risk for pneumonia. For workers in this industry, occupational injury is common, and preventative strategies have been developed over the years to mitigate such risks [29, 30]. However, bacterial infections, and especially pneumonia were not seen as risks for this industry before. The evidence delivered by this study calls for improved mitigation of pneumonia infection risk here as well, but more research on specific transmission routes is needed here to guide preventative strategies. The marine fisheries class did not show a similar pattern, with slightly lowered aHRs for both pneumonia infection and general bacterial infections. Although the nature of the work here is similar, access to healthcare might not be as easy for people working in this class due to prolonged periods of time they spend at sea; less accessible healthcare potentially leads to an underestimation of the risks for this group.

Specific aHRs for infections in the healthcare section showed that especially people working in residential care services and residential care for the elderly in Taiwan have a higher risk for pneumonia infections, respectively, around 3.49 and 1.75 times as high compared to the reference industrial classes. Although such settings are often referred to in relation to bacterial infections in research, this usually entails the elevated risks for people residing in such settings instead of people working there [31, 32]. This newly provided evidence suggests that infection control measures in residential care in Taiwan should be extended to protect people working here. Although infection prevention and control (IPC) strategies are proven to be effective in hospital settings, evidence of its effectiveness is lacking in literature for residential care facilities. Resources for IPC programs are vastly different between hospitals and residential care facilities, challenging effective implementation of such programs. Although residential care facilities might lack infectious disease experts or other resources such as diagnosis capacity, it has been reported that a combination of IPC measures endorsed by the World Health Organization is effective in reducing infection risk, starting with a stronger focus on education, monitoring and feedback on infections in such settings, followed by improved hygiene practices such as handwashing and the use of personal protective equipment. Improving the built environment of residential care facilities can also mitigate transmission and thereby infection risk [33]. Although the residential care classes can already start with implementing some of the simpler and less costly measures, additional risk assessments are needed to identify differences between residential care facilities in Taiwan. This could identify high-risk facilities for workers and residents and lessons can be learned from well performing facilities.

Also, within the healthcare section, workers in the social work services for children and youth were less likely to develop bacterial infections and pneumonia in this cohort. This can be partially explained by the less frequent contact and thereby transfer risk between workers here and their clientele, especially compared to their counterparts in residential care.

For people working in clinics in Taiwan, a higher general infection risk was found that contrasted with the lower risk found for people working in the hospital industrial class. This important finding suggests that infection control measures in hospital settings in Taiwan might be better implemented, compared to clinic settings. Improvements can, however, still be made in the hospital settings, as workers here still had a 1.2 times higher risk for pneumonia infections. Although clinics might not have the resources for infections control which hospitals have, lessons can be learned from the hospital setting. Increased awareness of preventative measures and transmission routes, can guide simple improvements in hygiene practices and personnel protection.

Despite Taiwan experiencing a relatively lower impact from COVID-19 in 2020, patients changed their healthcare-seeking behaviours, by avoiding healthcare settings [34, 35]. However, subsequent analyses excluding 2020 from the cohort revealed only a minimal effect of these changed dynamics on the risk of bacterial infections for healthcare workers.

This study’s unique approach to combine claim records from both NHI and Labor Insurance, provided a more comprehensive risk assessment for bacterial infections, and more specifically pneumonia, for certain industrial classes and especially compared to each other. By including over 10% of the complete Taiwanese population in this retrospective cohort, and over 28% of the working population, this big data approach has significant value for Taiwan, as it provides evidence for improvements in infection prevention for worker protection in industrial classes pointed out in this study. Preventing bacterial infections is crucial for individuals to improve their economic participation and to disrupt bacterial transmission chains, which is essential in combating bacterial AMR.

A limitation to this study is that ICD coding is not entirely objective, as healthcare providers must choose from a wide range of possibilities, often time pressured. For example, using a nonspecific ICD code, such as pneumonia due to an unspecified pathogen can be a misclassification if more specific coding was possible, leading to underreporting. In addition, practitioners can only record up to five ICD codes per diagnosis claim due to NHI registry design, so they prioritize based on severity. This can result in underreporting of bacterial infections when more severe conditions are present. These systemic biases likely affect Taiwan uniformly, causing misclassification and underreporting across all occupational classes in this study.

In addition, most labour insurance records before 2004 have not been digitized and were inaccessible. To address this, the study corrected the proportional hazards model with insurance duration as a parameter for time spent in an occupation. This approach is not ideal and likely underestimates the risk, as insurance duration slightly increased risks in the model. However, if employment duration data were available for a longer time span, it would increase the duration for all groups equally and thereby increasing the risk across all groups equally.

Finally, disparities in access to health services persist in Taiwan, contributing to higher premature mortality rates, for example, in less densely populated areas [36]. These underserved communities often rely on agricultural work, leading to underdiagnosis due to limited healthcare access. Consequently, this study likely underestimates the actual bacterial infection risks for agricultural workers. In addition, healthcare workers might be overrepresented in the data because they are more aware of and more likely to be diagnosed with bacterial infections. Despite this, the research shows that people working in hospital settings in Taiwan had a lower risk for general bacterial infections compared to reference industries. As this research is specific to Taiwan, a high-income country with robust infection control, its findings may not be easily generalizable to lower- and middle-income countries.

## Conclusion

This study revealed several occupational settings beyond the traditional healthcare scope in Taiwan that had significantly higher risks for bacterial infections, and especially pneumonia. This included workers in occupations related to soil, inland fishing and residential care, who need to be better protected from bacterial infection risks. The innovative methods used in this study to combine and analyse health insurance and labour insurance claim data, proved to be a powerful surveillance tool to assess occupational risks of bacterial infection. This approach could be further utilized in future surveillance to identify occupational risks of bacterial AMR.

## Supporting information

De Jong et al. supplementary material De Jong et al. supplementary material

## Data Availability

This study used restricted National Health Insurance and Labor Insurance claim data from Taiwan. Special permission was granted by the Ministry of Health and Welfare and the Institute of Labor and Occupational Safety, after National Taiwan University IRB approval. Strict measures apply to the access, data wrangling and analysis of these datasets, to ensure anonymization. All analysis was carried out in a secured datacentre run by the Ministry of Health and Welfare, export of these datasets is not allowed. Results of the analysis as reported in this manuscript could only be exported after careful examination and approval by the Ministry of Health and Welfare. Coding books and coding steps can be provided by the authors upon request.

## Supplementary material

For supplementary material accompanying this paper visit http://doi.org/10.1017/S0950268824001304. click here to view supplementary material
