# Supplementary material for: A nationwide cohort study on pneumonia infections among agriculture and healthcare workers in Taiwan
Source: Epidemiol Infect. 2024 Dec 5;152:e156. doi: 10.1017/S0950268824001304 (PMC11626447; doi:10.1017/S0950268824001304)
Supplement: De Jong et al. supplementary material [file S0950268824001304sup001.docx]

# Supplementing material

- **Supplement 1.** Mapped ICD codes for bacterial infections in Taiwan

## Supplement 1. Mapped ICD codes for bacterial infections in Taiwan

| ICD9CM | ICD9CM_full | ICD10CM | ICD10CM_full |
| --- | --- | --- | --- |
| 566 | Abscess of anal and rectal regions | K61.0 | Anal abscess |
| 566 | Abscess of anal and rectal regions | K61.1 | Rectal abscess |
| 566 | Abscess of anal and rectal regions | K61.2 | Anorectal abscess |
| 566 | Abscess of anal and rectal regions | K61.3 | Ischiorectal abscess |
| 566 | Abscess of anal and rectal regions | K61.4 | Intrasphincteric abscess |
| 513.0 | Abscess of lung | J85.0 | Gangrene and necrosis of lung |
| 513.0 | Abscess of lung | J85.1 | Abscess of lung with pneumonia |
| 513.0 | Abscess of lung | J85.2 | Abscess of lung without pneumonia |
| 513.1 | Abscess of mediastinum | J85.3 | Abscess of mediastinum |
| 510.0 | Empyema, with fistula | J86.0 | Pyothorax with fistula |
| 510.9 | Empyema, without mention of fistula | J86.9 | Pyothorax without fistula |
| 706.1 | Other acne | L70.0 | Acne vulgaris |
| 706.1 | Other acne | L70.1 | Acne conglobata |
| 706.0 | Acne varioliformis | L70.2 | Acne varioliformis |
| 706.1 | Other acne | L70.3 | Acne tropica |
| 706.1 | Other acne | L70.4 | Infantile acne |
| 706.1 | Other acne | L70.5 | Acne excoriee des jeunes filles |
| 706.1 | Other acne | L70.8 | Other acne |
| 706.1 | Other acne | L70.9 | Acne, unspecified |
| 540.0 | Acute appendicitis, with generalized peritonitis | K35.2 | Acute appendicitis with generalized peritonitis |
| 540.1 | Acute appendictis, with peritoneal abscess | K35.3 | Acute appendicitis with localized peritonitis |
| 466.11 | Acute bronchiolitis due to respiratory syncytial virus(RSV) | J21.0 | Acute bronchiolitis due to respiratory syncytial virus |
| 466.19 | Acute bronchiolitis due to other infectious organisms | J21.1 | Acute bronchiolitis due to human metapneumovirus |
| 466.19 | Acute bronchiolitis due to other infectious organisms | J21.8 | Acute bronchiolitis due to other specified organisms |
| 466.19 | Acute bronchiolitis due to other infectious organisms | J21.9 | Acute bronchiolitis, unspecified |
| 466.0 | Acute bronchitis | J20.9 | Acute bronchitis, unspecified |
| 320.0 | Hemophilus meningitis | G00.0 | Hemophilus meningitis |
| 320.1 | Pneumococcal meningitis | G00.1 | Pneumococcal meningitis |
| 320.2 | Streptococcal meningitis | G00.2 | Streptococcal meningitis |
| 320.3 | Staphylococcal meningitis | G00.3 | Staphylococcal meningitis |
| 320.89 | Meningitis due to other specified bacteria | G00.8 | Other bacterial meningitis |
| 320.9 | Meningitis due to unspecified bacterium | G00.9 | Bacterial meningitis, unspecified |
| 324.0 | Intracranial abscess | G06.0 | Intracranial abscess and granuloma |
| 324.1 | Intraspinal abscess | G06.1 | Intraspinal abscess and granuloma |
| 324.9 | Intracranial and intraspinal abscess, unspecified site | G06.2 | Extradural and subdural abscess, unspecified |
| 482.0 | Pneumonia due to Klebsiella pneumoniae | J15.0 | Pneumonia due to Klebsiella pneumoniae |
| 482.1 | Pneumonia due to Pseudomonas | J15.1 | Pneumonia due to Pseudomonas |
| 482.32 | Pneumonia due to Streptococcus, Group B | J15.3 | Pneumonia due to streptococcus, group B |
| 482.39 | Pneumonia due to other Streptococcus | J15.4 | Pneumonia due to other streptococci |
| 482.82 | Pneumonia due to Escherichia coli ﹝E. coli﹞ | J15.5 | Pneumonia due to Escherichia coli |
| 482.83 | Pneumonia due to other gram-negative bacteria | J15.6 | Pneumonia due to other aerobic Gram-negative bacteria |
| 483.0 | Pneumonia due to Mycoplasma pneumoniae | J15.7 | Pneumonia due to Mycoplasma pneumoniae |
| 482.89 | Other specified bacteria | J15.8 | Pneumonia due to other specified bacteria |
| 482.9 | Bacterial pneumonia, unspecified | J15.9 | Unspecified bacterial pneumonia |
| 483.1 | Pneumonia due to Chlamydia | J16.0 | Chlamydial pneumonia |
| 483.8 | Pneumonia due to other specified organism | J16.8 | Pneumonia due to other specified infectious organisms |
| 485 | Bronchopneumonia, organism unspecified | J18.0 | Bronchopneumonia, unspecified organism |
| 481 | Pneumococcal pneumonia [streptococcus pneumoniae pneumonia] | J18.1 | Lobar pneumonia, unspecified organism |
| 514 | Pulmonary congestion and hypostasis | J18.2 | Hypostatic pneumonia, unspecified organism |
| 486 | Pneumonia, organism unspecified | J18.8 | Other pneumonia, unspecified organism |
| 486 | Pneumonia, organism unspecified | J18.9 | Pneumonia, unspecified organism |
| 020.0 | Bubonic plague | A20.0 | Bubonic plague |
| 020.1 | Cellulocutaneous plague | A20.1 | Cellulocutaneous plague |
| 020.5 | Pneumonic plague, unspecified | A20.2 | Pneumonic plague |
| 020.8 | Other specified types of plague | A20.3 | Plague meningitis |
| 020.2 | Septicemic plague | A20.7 | Septicemic plague |
| 020.8 | Other specified types of plague | A20.8 | Other forms of plague |
| 020.9 | Plague, unspecified | A20.9 | Plague, unspecified |
| 021.0 | Ulceroglandular tularemia | A21.0 | Ulceroglandular tularemia |
| 021.3 | Oculoglandular tularemia | A21.1 | Oculoglandular tularemia |
| 021.2 | Pulmonary tularemia | A21.2 | Pulmonary tularemia |
| 021.1 | Enteric tularemia | A21.3 | Gastrointestinal tularemia |
| 021.8 | Other specified tularemia | A21.7 | Generalized tularemia |
| 021.8 | Other specified tularemia | A21.8 | Other forms of tularemia |
| 021.9 | Unspecified tularemia | A21.9 | Tularemia, unspecified |
| 022.0 | Cutaneous anthrax | A22.0 | Cutaneous anthrax |
| 022.1 | Pulmonary anthrax | A22.1 | Pulmonary anthrax |
| 022.2 | Gastrointestinal anthrax | A22.2 | Gastrointestinal anthrax |
| 022.3 | Anthrax septicemia | A22.7 | Anthrax sepsis |
| 022.8 | Other specified manifestations of anthrax | A22.8 | Other forms of anthrax |
| 022.9 | Anthrax, unspecified | A22.9 | Anthrax, unspecified |
| 023.0 | Brucella melitensis | A23.0 | Brucellosis due to Brucella melitensis |
| 023.1 | Brucella abortus | A23.1 | Brucellosis due to Brucella abortus |
| 023.2 | Brucella suis | A23.2 | Brucellosis due to Brucella suis |
| 023.3 | Brucella canis | A23.3 | Brucellosis due to Brucella canis |
| 023.8 | Other brucellosis | A23.8 | Other brucellosis |
| 023.9 | Brucellosis, unspecified | A23.9 | Brucellosis, unspecified |
| 024 | Glanders | A24.0 | Glanders |
| 025 | Melioidosis | A24.1 | Acute and fulminating melioidosis |
| 025 | Melioidosis | A24.2 | Subacute and chronic melioidosis |
| 025 | Melioidosis | A24.3 | Other melioidosis |
| 026.0 | Spirillary fever | A25.0 | Spirillosis |
| 026.1 | Streptobacillary fever | A25.1 | Streptobacillosis |
| 026.9 | Unspecified rat-bite fever | A25.9 | Rat-bite fever, unspecified |
| 027.1 | Erysipelothrix infection | A26.0 | Cutaneous erysipeloid |
| 027.1 | Erysipelothrix infection | A26.7 | Erysipelothrix sepsis |
| 027.1 | Erysipelothrix infection | A26.8 | Other forms of erysipeloid |
| 027.1 | Erysipelothrix infection | A26.9 | Erysipeloid, unspecified |
| 100.0 | Leptospirosis icterohemorrhagica | A27.0 | Leptospirosis icterohemorrhagica |
| 100.9 | Leptospirosis,unspecified | A27.9 | Leptospirosis, unspecified |
| 027.2 | Pasteurellosis | A28.0 | Pasteurellosis |
| 078.3 | Cat-scratch disease | A28.1 | Cat-scratch disease |
| 027.8 | Other specified zoonotic bacterial diseases | A28.2 | Extraintestinal yersiniosis |
| 027.8 | Other specified zoonotic bacterial diseases | A28.8 | Other specified zoonotic bacterial diseases, not elsewhere classified |
| 027.9 | Unspecified zoonotic bacterial disease | A28.9 | Zoonotic bacterial disease, unspecified |
| 574.00 | Calculus of gallbladder with acute cholecystitis, without mention of obstruction | K80.00 | Calculus of gallbladder with acute cholecystitis without obstruction |
| 574.01 | Calculus of gallbladder with acute cholecystitis with obstruction | K80.01 | Calculus of gallbladder with acute cholecystitis with obstruction |
| 574.10 | Calculus of gallbladder with other cholecystitis, without mention of obstruction | K80.10 | Calculus of gallbladder with chronic cholecystitis without obstruction |
| 574.11 | Calculus of gallbladder with other cholecystitis with obstruction | K80.11 | Calculus of gallbladder with chronic cholecystitis with obstruction |
| 574.50 | Calculus of bile duct without mention of cholecystitis without mention of obstruction | K80.30 | Calculus of bile duct with cholangitis, unspecified, without obstruction |
| 574.51 | Calculus of bile duct without mention of cholecystitis with obstruction | K80.31 | Calculus of bile duct with cholangitis, unspecified, with obstruction |
| 574.30 | Calculus of bile duct with acute cholecystitis, without mention of obstruction | K80.40 | Calculus of bile duct with cholecystitis, unspecified, without obstruction |
| 574.31 | Calculus of bile duct with acute cholecystitis, with obstruction | K80.41 | Calculus of bile duct with cholecystitis, unspecified, with obstruction |
| 473.0 | Chronic sinusitis of maxillary | J32.0 | Chronic maxillary sinusitis |
| 473.1 | Chronic sinusitis of frontal | J32.1 | Chronic frontal sinusitis |
| 473.2 | Chronic sinusitis of ethmoidal | J32.2 | Chronic ethmoidal sinusitis |
| 473.3 | Chronic sinusitis of sphenoidal | J32.3 | Chronic sphenoidal sinusitis |
| 473.8 | Other chronic sinusitis | J32.4 | Chronic pansinusitis |
| 473.8 | Other chronic sinusitis | J32.8 | Other chronic sinusitis |
| 473.9 | Unspecified sinusitis (chronic) | J32.9 | Chronic sinusitis, unspecified |
| 090.1 | Early congenital syphilis, latent | A50.1 | Early congenital syphilis, latent |
| 090.2 | Early congenital syphilis, unspecified | A50.2 | Early congenital syphilis, unspecified |
| 090.6 | Late congenital syphilis, latent | A50.6 | Late congenital syphilis, latent |
| 090.7 | Late congenital syphilis, unspecified | A50.7 | Late congenital syphilis, unspecified |
| 090.9 | Congenital syphilis, unspecified | A50.9 | Congenital syphilis, unspecified |
| 091.0 | Genital syphilis (primary) | A51.0 | Primary genital syphilis |
| 091.1 | Primary anal syphilis | A51.1 | Primary anal syphilis |
| 091.2 | Other primary syphilis | A51.2 | Primary syphilis of other sites |
| 092.9 | Early syphilis, latent, unspecified | A51.5 | Early syphilis, latent |
| 094.3 | Asymptomatic neurosyphilis | A52.2 | Asymptomatic neurosyphilis |
| 094.9 | Neurosyphilis, unspecified | A52.3 | Neurosyphilis, unspecified |
| 096 | Late syphilis, latent | A52.8 | Late syphilis, latent |
| 097.0 | Late syphilis, unspecified | A52.9 | Late syphilis, unspecified |
| 097.1 | Latent syphilis, unspecified | A53.0 | Latent syphilis, unspecified as early or late |
| 097.9 | Syphilis, unspecified | A53.9 | Syphilis, unspecified |
| 098.0 | Acute gonococcal infections of lower genitourinary tract | A54.1 | Gonococcal infection of lower genitourinary tract with periurethral and accessory gland abscess |
| 098.6 | Gonococcal infection of pharynx | A54.5 | Gonococcal pharyngitis |
| 098.7 | Gonococcal infection of anus and rectum | A54.6 | Gonococcal infection of anus and rectum |
| 099.55 | Unspecified genitourinary site venereal disease due to Chlamydia trachomatis | A56.2 | Chlamydial infection of genitourinary tract, unspecified |
| 099.52 | Anus and rectum venereal disease due to Chlamydia trachomatis | A56.3 | Chlamydial infection of anus and rectum |
| 099.51 | Pharynx venereal disease due to Chlamydia trachomatis | A56.4 | Chlamydial infection of pharynx |
| 099.59 | Other specified site venereal disease due to Chlamydia trachomatis | A56.8 | Sexually transmitted chlamydial infection of other sites |
| 131.8 | Trichomoniasis of other specified sites | A59.8 | Trichomoniasis of other sites |
| 131.9 | Trichomoniasis, unspecified | A59.9 | Trichomoniasis, unspecified |
| 054.10 | Genital herpes, unspecified | A60.9 | Anogenital herpesviral infection, unspecified |
| 099.8 | Other specified venereal diseases | A63.8 | Other specified predominantly sexually transmitted diseases |
| 421.0 | Acute and subacute bacterial endocarditis | I33.0 | Acute and subacute infective endocarditis |
| 421.9 | Acute endocarditis, unspecified | I33.9 | Acute and subacute endocarditis, unspecified |
| 728.0 | Infective myositis | M60.08 | Infective myositis, other site |
| 728.0 | Infective myositis | M60.09 | Infective myositis, multiple sites |
| 727.89 | Other disorders of synovium, tendon, and bursa | M65.00 | Abscess of tendon sheath, unspecified site |
| 727.89 | Other disorders of synovium, tendon, and bursa | M65.08 | Abscess of tendon sheath, other site |
| 727.09 | Other synovitis and tenosynovitis | M65.18 | Other infective (teno)synovitis, other site |
| 727.09 | Other synovitis and tenosynovitis | M65.19 | Other infective (teno)synovitis, multiple sites |
| 727.89 | Other disorders of synovium, tendon, and bursa | M71.00 | Abscess of bursa, unspecified site |
| 727.89 | Other disorders of synovium, tendon, and bursa | M71.08 | Abscess of bursa, other site |
| 727.89 | Other disorders of synovium, tendon, and bursa | M71.09 | Abscess of bursa, multiple sites |
| 727.3 | Other bursitis | M71.10 | Other infective bursitis, unspecified site |
| 727.3 | Other bursitis | M71.18 | Other infective bursitis, other site |
| 727.3 | Other bursitis | M71.19 | Other infective bursitis, multiple sites |
| 730.28 | Unspecified osteomyelitis, other specified sites | M46.20 | Osteomyelitis of vertebra, site unspecified |
| 730.28 | Unspecified osteomyelitis, other specified sites | M46.22 | Osteomyelitis of vertebra, cervical region |
| 730.28 | Unspecified osteomyelitis, other specified sites | M46.25 | Osteomyelitis of vertebra, thoracolumbar region |
| 730.28 | Unspecified osteomyelitis, other specified sites | M46.28 | Osteomyelitis of vertebra, sacral and sacrococcygeal region |
| 722.90 | Unspecified disc disorder | M46.40 | Discitis, unspecified, site unspecified |
| 722.91 | Cervical intervertebral disc disorder | M46.42 | Discitis, unspecified, cervical region |
| 722.91 | Cervical intervertebral disc disorder | M46.43 | Discitis, unspecified, cervicothoracic region |
| 722.92 | Thoracic intervertebral disc disorder | M46.44 | Discitis, unspecified, thoracic region |
| 722.92 | Thoracic intervertebral disc disorder | M46.45 | Discitis, unspecified, thoracolumbar region |
| 722.93 | Lumbar intervertebral disc disorder | M46.46 | Discitis, unspecified, lumbar region |
| 722.93 | Lumbar intervertebral disc disorder | M46.47 | Discitis, unspecified, lumbosacral region |
| 722.90 | Unspecified disc disorder | M46.49 | Discitis, unspecified, multiple sites in spine |
| 720.89 | Other inflammatory spondylopathies | M46.50 | Other infective spondylopathies, site unspecified |
| 720.89 | Other inflammatory spondylopathies | M46.51 | Other infective spondylopathies, occipito-atlanto-axial region |
| 720.89 | Other inflammatory spondylopathies | M46.52 | Other infective spondylopathies, cervical region |
| 720.89 | Other inflammatory spondylopathies | M46.53 | Other infective spondylopathies, cervicothoracic region |
| 720.89 | Other inflammatory spondylopathies | M46.54 | Other infective spondylopathies, thoracic region |
| 720.89 | Other inflammatory spondylopathies | M46.55 | Other infective spondylopathies, thoracolumbar region |
| 720.89 | Other inflammatory spondylopathies | M46.56 | Other infective spondylopathies, lumbar region |
| 720.89 | Other inflammatory spondylopathies | M46.57 | Other infective spondylopathies, lumbosacral region |
| 720.89 | Other inflammatory spondylopathies | M46.58 | Other infective spondylopathies, sacral and sacrococcygeal region |
| 720.89 | Other inflammatory spondylopathies | M46.59 | Other infective spondylopathies, multiple sites in spine |
| 030.2 | Indeterminate﹝group I﹞ | A30.0 | Indeterminate leprosy |
| 030.1 | Tuberculoid﹝type T﹞ | A30.1 | Tuberculoid leprosy |
| 030.3 | Borderline﹝group B﹞ | A30.2 | Borderline tuberculoid leprosy |
| 030.3 | Borderline﹝group B﹞ | A30.3 | Borderline leprosy |
| 030.3 | Borderline﹝group B﹞ | A30.4 | Borderline lepromatous leprosy |
| 030.0 | Lepromatous﹝type L﹞ | A30.5 | Lepromatous leprosy |
| 030.8 | Other specified leprosy | A30.8 | Other forms of leprosy |
| 030.9 | Leprosy, unspecified | A30.9 | Leprosy, unspecified |
| 031.0 | Pulmonary diseases due to other mycobacteria | A31.0 | Pulmonary mycobacterial infection |
| 031.1 | Cutaneous diseases due to other mycobacteria | A31.1 | Cutaneous mycobacterial infection |
| 031.2 | Disseminated diseases due to other mycobacteria | A31.2 | Disseminated mycobacterium avium-intracellulare complex (DMAC) |
| 031.8 | Other specified mycobacteria diseases | A31.8 | Other mycobacterial infections |
| 031.9 | Unspecified diseases due to mycobacteria | A31.9 | Mycobacterial infection, unspecified |
| 027.0 | Listeriosis | A32.0 | Cutaneous listeriosis |
| 027.0 | Listeriosis | A32.7 | Listerial sepsis |
| 027.0 | Listeriosis | A32.9 | Listeriosis, unspecified |
| 032.0 | Faucial diphtheria | A36.0 | Pharyngeal diphtheria |
| 032.1 | Nasopharyngeal diphtheria | A36.1 | Nasopharyngeal diphtheria |
| 032.3 | Laryngeal diphtheria | A36.2 | Laryngeal diphtheria |
| 032.85 | Cutaneous diphtheria | A36.3 | Cutaneous diphtheria |
| 032.9 | Diphtheria, unspecified | A36.9 | Diphtheria, unspecified |
| 036.0 | Meningococcal meningitis | A39.0 | Meningococcal meningitis |
| 036.3 | Waterhouse-Friderichsen syndrome, meningococcal | A39.1 | Waterhouse-Friderichsen syndrome |
| 036.2 | Meningococcemia | A39.2 | Acute meningococcemia |
| 036.2 | Meningococcemia | A39.3 | Chronic meningococcemia |
| 036.2 | Meningococcemia | A39.4 | Meningococcemia, unspecified |
| 036.9 | Meningococcal infection, unspecified | A39.9 | Meningococcal infection, unspecified |
| 041.5 | Hemophilus influenzae﹝H. influenzae﹞infections of unspecified site | A49.2 | Hemophilus influenzae infection, unspecified site |
| 041.81 | Mycoplasma infections of unspecified site | A49.3 | Mycoplasma infection, unspecified site |
| 041.89 | Other specified bacterial infections of unspecified site | A49.8 | Other bacterial infections of unspecified site |
| 041.9 | Unspecified bacterial infection of unspecified site | A49.9 | Bacterial infection, unspecified |
| 080 | Louse-borne﹝epidemic﹞typhus | A75.0 | Epidemic louse-borne typhus fever due to Rickettsia prowazekii |
| 081.1 | Brill's disease | A75.1 | Recrudescent typhus [Brill's disease] |
| 081.0 | Murine﹝endemic﹞typhus | A75.2 | Typhus fever due to Rickettsia typhi |
| 081.2 | Scrub typhus | A75.3 | Typhus fever due to Rickettsia tsutsugamushi |
| 081.9 | Typhus, unspecified | A75.9 | Typhus fever, unspecified |
| 083.1 | Trench fever | A79.0 | Trench fever |
| 083.2 | Rickettsialpox | A79.1 | Rickettsialpox due to Rickettsia akari |
| 083.9 | Rickettsiosis, unspecified | A79.9 | Rickettsiosis, unspecified |
| 076.0 | Trachoma, initial stage | A71.0 | Initial stage of trachoma |
| 076.1 | Trachoma, active stage | A71.1 | Active stage of trachoma |
| 076.9 | Trachoma, unspecified | A71.9 | Trachoma, unspecified |
| 077.98 | Unspecified diseases of conjunctiva due to Chlamydiae | A74.0 | Chlamydial conjunctivitis |
| 102.0 | Initial lesions of yaws | A66.0 | Initial lesions of yaws |
| 102.1 | Multiple papillomata and wet crab yaws | A66.1 | Multiple papillomata and wet crab yaws |
| 102.2 | Other early skin lesions of yaws | A66.2 | Other early skin lesions of yaws |
| 102.3 | Hyperkeratosis of yaws | A66.3 | Hyperkeratosis of yaws |
| 102.4 | Gummata and ulcers of yaws | A66.4 | Gummata and ulcers of yaws |
| 102.5 | Gangosa | A66.5 | Gangosa |
| 102.6 | Bone and joint lesions of yaws | A66.6 | Bone and joint lesions of yaws |
| 102.7 | Other manifestations of yaws | A66.7 | Other manifestations of yaws |
| 102.8 | Latent yaws | A66.8 | Latent yaws |
| 102.9 | Yaws, unspecified | A66.9 | Yaws, unspecified |
| 103.0 | Primary lesions of pinta (carate) | A67.0 | Primary lesions of pinta |
| 103.1 | Intermediate lesions of pinta (carate) | A67.1 | Intermediate lesions of pinta |
| 103.2 | Late lesions of pinta (carate) | A67.2 | Late lesions of pinta |
| 103.3 | Mixed lesions of pinta (carate) | A67.3 | Mixed lesions of pinta |
| 103.9 | Pinta, unspecified | A67.9 | Pinta, unspecified |
| 087.0 | Louse-borne relapsing fever | A68.0 | Louse-borne relapsing fever |
| 087.1 | Tick-borne relapsing fever | A68.1 | Tick-borne relapsing fever |
| 087.9 | Relapsing fever, unspecified | A68.9 | Relapsing fever, unspecified |
| 101 | Vincent's angina | A69.0 | Necrotizing ulcerative stomatitis |
| 101 | Vincent's angina | A69.1 | Other Vincent's infections |
| 104.8 | Other specified spirochetal infections | A69.8 | Other specified spirochetal infections |
| 104.9 | Spirochetal infection, unspecified | A69.9 | Spirochetal infection, unspecified |
| 615.0 | Acute inflammatory diseases of uterus, except cervix | N71.0 | Acute inflammatory disease of uterus |
| 615.1 | Chronic inflammatory diseases of uterus, except cervix | N71.1 | Chronic inflammatory disease of uterus |
| 615.9 | Unspecified inflammatory disease of uterus | N71.9 | Inflammatory disease of uterus, unspecified |
| 614.3 | Acute parametritis and pelvic cellulitis | N73.0 | Acute parametritis and pelvic cellulitis |
| 614.4 | Chronic or unspecified parametritis and pelvic cellulitis | N73.1 | Chronic parametritis and pelvic cellulitis |
| 614.4 | Chronic or unspecified parametritis and pelvic cellulitis | N73.2 | Unspecified parametritis and pelvic cellulitis |
| 614.5 | Acute or unspecified pelvic peritonitis, female | N73.3 | Female acute pelvic peritonitis |
| 614.7 | Other chronic pelvic peritonitis, female | N73.4 | Female chronic pelvic peritonitis |
| 614.6 | Pelvic peritoneal adhesions, female（postoperative）(postinfection) | N73.6 | Female pelvic peritoneal adhesions (postinfective) |
| 614.8 | Other specified inflammatory disease of female pelvic organs and tissues | N73.8 | Other specified female pelvic inflammatory diseases |
| 614.9 | Unspecified inflammatory disease of female pelvic organs and tissues | N73.9 | Female pelvic inflammatory disease, unspecified |
| 567.2 | Other suppurative peritonitis | K65.0 | Generalized (acute) peritonitis |
| 567.8 | Other specified peritonitis | K65.8 | Other peritonitis |
| 567.9 | Unspecified peritonitis | K65.9 | Peritonitis, unspecified |
| 601.0 | Acute prostatitis | N41.0 | Acute prostatitis |
| 601.1 | Chronic prostatitis | N41.1 | Chronic prostatitis |
| 601.2 | Abscess of prostate | N41.2 | Abscess of prostate |
| 601.3 | Prostatocystitis | N41.3 | Prostatocystitis |
| 601.8 | Other specified inflammatory diseases of prostate | N41.4 | Granulomatous prostatitis |
| 601.8 | Other specified inflammatory diseases of prostate | N41.8 | Other inflammatory diseases of prostate |
| 601.9 | Prostatitis, unspecified | N41.9 | Inflammatory disease of prostate, unspecified |
| 012.16 | Tuberculosis of intrathoracic lymph nodes, tubercle bacilli not found by bacteriological or histological examination but tuberculosis confirmed by other methods [inoculation of animals] | A15.4 | Tuberculosis of intrathoracic lymph nodes |
| 012.26 | Isolated tracheal or bronchial tuberculosis , tubercle bacilli not found by bacteriological or histological examination but tuberculosis confirmed by other methods [inoculation of animals] | A15.5 | Tuberculosis of larynx, trachea and bronchus |
| 012.06 | Tuberculous pleurisy , tubercle bacilli not found by bacteriological or histological examination but tuberculosis confirmed by other methods [inoculation of animals] | A15.6 | Tuberculous pleurisy |
| 010.90 | Primary tuberculous infection, unspecified | A15.7 | Primary respiratory tuberculosis |
| 012.86 | Other specified respiratory tuberculosis, tubercle bacilli not found by bacteriological or histological examination but tuberculosis confirmed by other methods [inoculation of animals] | A15.8 | Other respiratory tuberculosis |
| 013.00 | Tuberculous meningitis, unspecified | A17.0 | Tuberculous meningitis |
| 013.10 | Tuberculoma of meninges, unspecified | A17.1 | Meningeal tuberculoma |
| 013.90 | Unspecified tuberculosis of central nervous system , unspecified | A17.9 | Tuberculosis of nervous system, unspecified |
| 017.20 | Tuberculosis of peripheral lymph nodes, unspecified | A18.2 | Tuberculous peripheral lymphadenopathy |
| 017.00 | Tuberculosis of skin and subcutaneous cellular tissue, unspecified | A18.4 | Tuberculosis of skin and subcutaneous tissue |
| 017.40 | Tuberculosis of ear, unspecified | A18.6 | Tuberculosis of (inner) (middle) ear |
| 017.60 | Tuberculosis of adrenal glands, unspecified | A18.7 | Tuberculosis of adrenal glands |
| 018.00 | Acute miliary tuberculosis, unspecified | A19.0 | Acute miliary tuberculosis of a single specified site |
| 018.00 | Acute miliary tuberculosis, unspecified | A19.1 | Acute miliary tuberculosis of multiple sites |
| 018.00 | Acute miliary tuberculosis, unspecified | A19.2 | Acute miliary tuberculosis, unspecified |
| 018.80 | Other specified miliary tuberculosis, unsepcified | A19.8 | Other miliary tuberculosis |
| 018.90 | Miliary tuberculosis, unspecified | A19.9 | Miliary tuberculosis, unspecified |
| 597.0 | Urethral abscess | N34.0 | Urethral abscess |
| 099.40 | Other unspecified nongonococcal urethritis | N34.1 | Nonspecific urethritis |
| 597.89 | Other urethritis | N34.2 | Other urethritis |
| 597.81 | Urethral syndrome NOS | N34.3 | Urethral syndrome, unspecified |
